# Human tumor microenvironment chip evaluates the consequences of platelet extravasation and combinatorial antitumor-antiplatelet therapy in ovarian cancer

**DOI:** 10.1126/sciadv.abg5283

**Published:** 2021-07-21

**Authors:** Biswajit Saha, Tanmay Mathur, James J. Tronolone, Mithil Chokshi, Giriraj K. Lokhande, Amirali Selahi, Akhilesh K. Gaharwar, Vahid Afshar-Kharghan, Anil K. Sood, Gang Bao, Abhishek Jain

**Affiliations:** 1 Department of Biomedical Engineering, College of Engineering, Texas A&M University, College Station, TX 77840, USA.; 2 Department of Bioengineering, George R. Brown School of Engineering, Rice University, Houston, TX 77005, USA.; 3 Materials Science and Engineering, College of Engineering, Texas A&M University, College Station, TX 77840, USA.; 4 Center for Remote Health Technologies and Systems, Texas A&M University, College Station TX 77840, USA.; 5 Department of Benign Hematology, The University of Texas MD Anderson Cancer Center, Houston, TX 77030, USA.; 6 Department of Gynecologic Oncology and Reproductive Medicine, The University of Texas MD Anderson Cancer Center, Houston, TX 77030, USA.; 7 Department of Medical Physiology, College of Medicine, Texas A&M Health Science Center, Bryan, TX 77807, USA.; 8 Department of Cardiovascular Sciences, Houston Methodist Academic Institute, Houston, TX 77030, USA.

## Abstract

Platelets extravasate from the circulation into tumor microenvironment, enable metastasis, and confer resistance to chemotherapy in several cancers. Therefore, arresting tumor-platelet cross-talk with effective and atoxic antiplatelet agents in combination with anticancer drugs may serve as an effective cancer treatment strategy. To test this concept, we create an ovarian tumor microenvironment chip (OTME-Chip) that consists of a platelet-perfused tumor microenvironment and which recapitulates platelet extravasation and its consequences. By including gene-edited tumors and RNA sequencing, this organ-on-chip revealed that platelets and tumors interact through glycoprotein VI (GPVI) and tumor galectin-3 under shear. Last, as proof of principle of a clinical trial, we showed that a GPVI inhibitor, Revacept, impairs metastatic potential and improves chemotherapy. Since GPVI is an antithrombotic target that does not impair hemostasis, it represents a safe cancer therapeutic. We propose that OTME-Chip could be deployed to study other vascular and hematological targets in cancer.

## Teaser

A tumor microenvironment chip reveals how platelets may fuel cancer metastasis and chemoresistance and unravels a new treatment.

## INTRODUCTION

Platelets have long been recognized as anuclear blood constituents that regulate hemostasis and thrombosis. Platelets are also the first responders in the pathobiology of cancer: They play both structural and functional roles as reporters and transporters within the tumor-vascular organ (*1*, *2*). To date, in vivo systems have been widely used in our understanding of cancer metastasis and its control through platelet function. For example, both mouse models (*3*–*5*) and human clinical studies of ovarian cancer (*6*) have shown that platelets extravasate into the solid tumor microenvironment and facilitate proliferation. A few animal studies have proposed the link between platelet extravasation and metastasis in ovarian cancer (*5*, *7*). Some reports also indicate that platelet surface receptor GPVI interacts with circulating tumor cell surface glycoprotein galectin-3 and potentiates its growth (*7*–*13*). However, a more detailed understanding of pathophysiological outcomes of transendothelial platelet extravasation in cancer remains limited. Whether extravasated platelets can affect primary solid tumors through their GPVI remains questionable. In addition, because vascular shear regulates platelet GPVI activation (*14*), it remains to be tested whether shear can also regulate platelet interaction with cancer cells (*15*). Extravasated platelets are also known to release tumor-promoting growth factors and cytokines/interleukins (ILs) (*5*, *7*) and induce chemoresistance in in vivo models (*9*, *16*). Therefore, antiplatelet therapeutics in cancer offered along with chemotherapy can be helpful. However, while the in vivo models have provided foundational knowledge of platelet interactions with cancer cells, new preclinical models are still needed that can provide a reductionist approach to systematically investigating tumor-platelet-drug cross-talk.

Microphysiological models, also termed organ-on-chip platforms, are particularly well suited for mimicking longitudinal cancer events and preclinical drug discovery as they offer a bottoms-up (simple to incrementally complex) and dissectible approach in biological system design, define tumor microenvironment and three-dimensional (3D) architecture, and enable imaging and molecular analyses with high spatiotemporal resolution. Now, there is an unmet need for in vitro microphysiological models that can integrate the analysis of vascular and blood compartments to that of the tumor microenvironment. Studies with these models can be conducted using exclusively human-derived primary tumor and blood cells or their CRISPR gene-edited counterparts. In prior studies, we have demonstrated the utility of organ-on-chip for faithfully modeling vascular dysfunction and platelet hyperactivity (*17*–*19*). More recently, we demonstrated that ovarian cancer-on-chip (termed OvCa-Chip) reproduces platelet extravasation from the blood vessel into tumors (*20*). However, our prior design could not analyze the after platelet extravasation events that potentially can increase cancer cell proliferation, induce chemoresistance, and enhance metastasis. This was partly because OvCa-Chip lacked the technological capability of including a tumor microenvironment that can visualize and analyze invasion dynamics.

Addressing this critical deficiency here, we engineered an ovarian tumor microenvironment organ-on-chip platform (OTME-Chip), which, in addition to the tumors interfacing platelet-perfused vascular endothelial tissue, also incorporates an adjacent well-defined collagen hydrogel–based extracellular matrix (ECM) microenvironment. This novel integration of a highly organized hydrogel architecture adjacent to the tumor cell chamber enables precise visualization of cancer cell invasion dynamics in response to the biophysical and biological effects of platelets extravasated through the endothelium into the tumor microenvironment. Using CRISPR-Cas9 knockout (KO) cancer cells, our platform revealed that platelets might promote ovarian cancer metastasis and chemoresistance through a shear-dependent interaction of their GPVI with galectin-3 expressed on the cancer cells. Last, we explored a novel treatment opportunity to arrest ovarian cancer metastasis and increase the effects of chemotherapy via an antiplatelet drug that is relatively safe and currently undergoing clinical trials for other disease conditions. These results were further validated by performing next-generation sequencing and subsequent differential gene expression analysis of platelet-interacted cancer cells extracted from the OTME-Chip. Together, our work suggests that OTME-Chip, integrated with gene editing and next-generation RNA sequencing (RNA-seq) tools, is a platform to advance the discovery of novel antiplatelet therapeutics against tumor metastasis and chemoresistance.

## RESULTS

### Formation of OTME-Chip

**Fig. 1:**
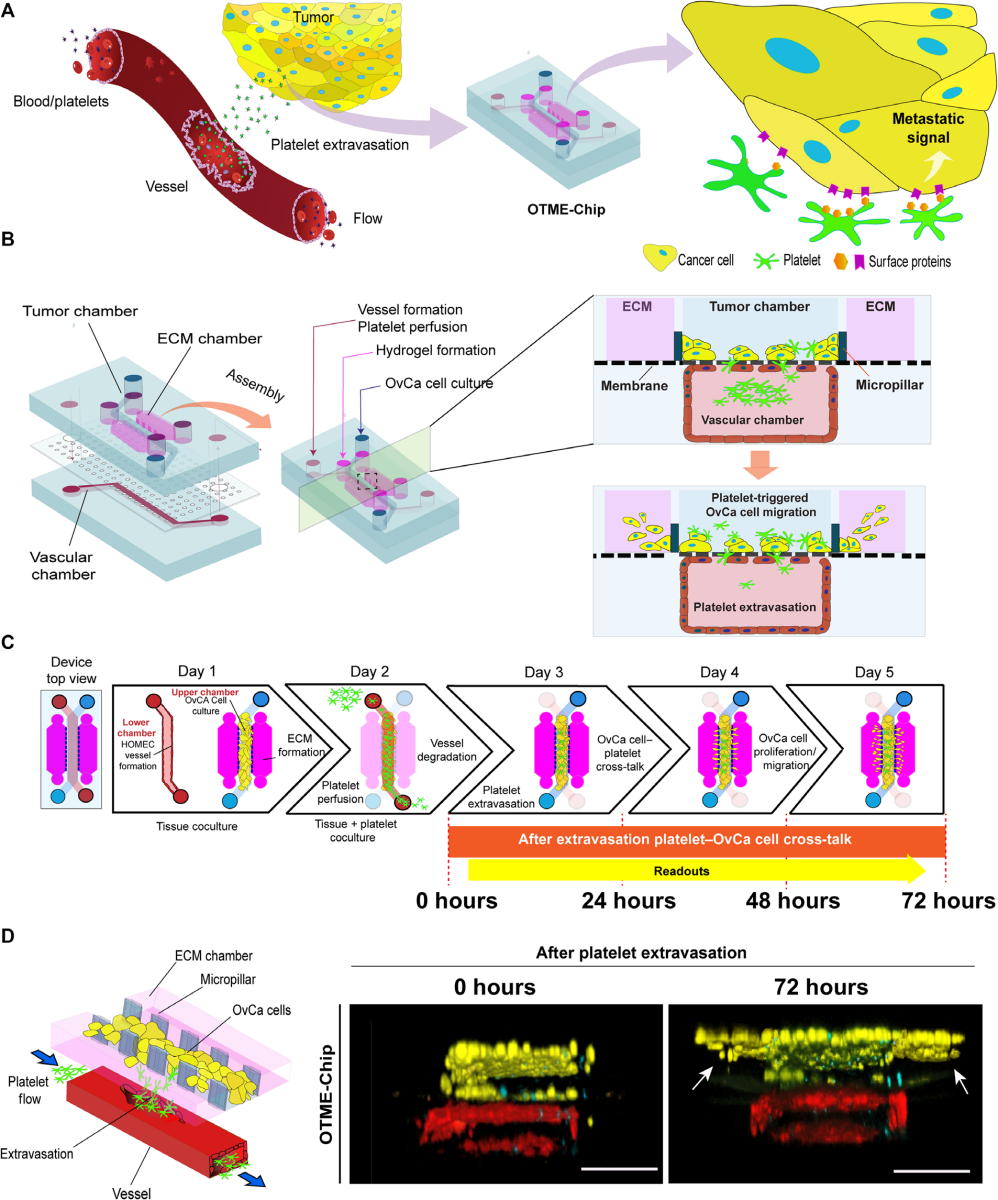
Microengineering of the OTME-Chip. (**A**) A conceptual infographic of the human tumor microenvironment showing that the cancer cells interact with the neighboring blood vessels, making them permeable, and recruiting platelets into their vicinity. These extravasated platelets actively bind their ligands to the tumor surface receptors and result in tumor proliferation and chemoresistance, which can potentially be arrested by antiplatelet drugs. (**B**) Engineering drawing of the microdevice containing two PDMS compartments separated by a thin porous membrane that reproduces the microarchitecture of the tumor-vascular interface (left). On the right, cross-sectional side view of the OTME-Chip describes tissue organization inside the chip. (**C**) Illustration showing the timeline and steps of OTME-Chip formation, platelet extravasation into the tumors, and following consequences. Cancer cell dynamics and molecular readouts are analyzed every 24 hours after platelet extravasation. (**D**) Schematic diagram of OTME-Chip that shows tumor invasion dynamics can be systematically visualized and characterized after platelet extravasation from the bottom vascular chamber into the top tumor chamber. On the right, cross-sectional side view of 3D confocal scan of OTME-Chip showing cancer cells (yellow), endothelial cells (red), and platelets (cyan) at 0 hours (left) and 72 hours (right) after platelet extravasation. Scale bars, 100 μm.

We were inspired to design an organ-chip microfluidic platform that served as a 3D organomimetic in vitro model of the tumor microenvironment, interfaced with a blood vessel and flow of platelets (Fig. 1A). Here, our primary goal was to enable the investigation of cancer cell–platelet dynamics after platelets extravasate through the endothelium into the ovarian tumors. We kept a few elements of the existing platform of our recently published OvCa-Chip—a two-chamber organ-on-chip technology consisting of cancer cells cocultured with a 3D endothelial vessel, separated by a matrix-coated polydimethylsiloxane (PDMS) membrane (see Materials and Methods for details)—but to enable longitudinal studies of cancer progression and to analyze the effect of extravasated platelets on cancer cell proliferation and invasiveness, we completely reengineered the top tumor chamber of the device by adding two adjacent ECM chambers separated by an array of PDMS micropillars on the sides (Fig. 1B). This biosystem design strategy, now termed OTME-Chip, was inspired by a different organ-on-a-chip technology that modeled metastatic invasiveness of colorectal and breast cancer cells through artificially created ECM-mimicking materials (*21*, *22*). This design does not permit blood perfusion or an intimate analysis of the interactions between the cancer cells, vascular cells, and blood constituents. However, in the OTME-Chip, the fusion of OvCa-Chip to this pillar structure design provides us the first opportunity to investigate the critical platelet–cancer cell interactions under flow, while still keeping the technology relatively simple and easy to adapt. First, we established the ECM compartments by injecting a pregel solution made of collagen type I and incubated the chip to form a semisolid hydrogel limited by micropillars. Next, we seeded the lower chamber of the device with human ovarian microvascular endothelial cells (HOMECs) to form an intact 3D vascular lumen. This was followed by the seeding of A2780 epithelial ovarian cancer cells in the central region of the top tumor chamber that formed the interface of a blood vessel and ovarian tumor tissue with the ECM. Further, we sustained this coculture with perfusion of freshly derived platelets from human blood inside the vascular lumen under microvascular shear for 3 days. Within 3 days, platelets increasingly extravasate into the ovarian cancer cells through the endothelium within the device (*20*).

After the platelets have extravasated into the tumor chamber, we now focused on new analyses on platelet interactions with cancer cells and consequences. We further progressed the coculture of the ovarian cancer cells with extravasated platelets together inside OTME-Chip for another 3 days with medium perfusion that is typical of ovarian interstitial flow (~0.5 to 1 dyne cm^−2^) (*23*). We then performed cellular and molecular analytics every 24 hours (Fig. 1C). After platelet extravasation from the vessel into the tumor compartment, we observed invasion of cancer cells through the pillars into the laterally created hydrogel ECM in the OTME-Chip (Fig. 1D). In contrast, no such invasion by cancer cells within ECM was detected when they were kept devoid of platelets inside the tumor chamber (Control-Chip). Therefore, these data support the ability of our platform to model platelet-tumor effects within the tumor microenvironment.

### Platelets interact with cancer cells through binding of GPVI to galectin-3 under shear

**Fig. 2:**
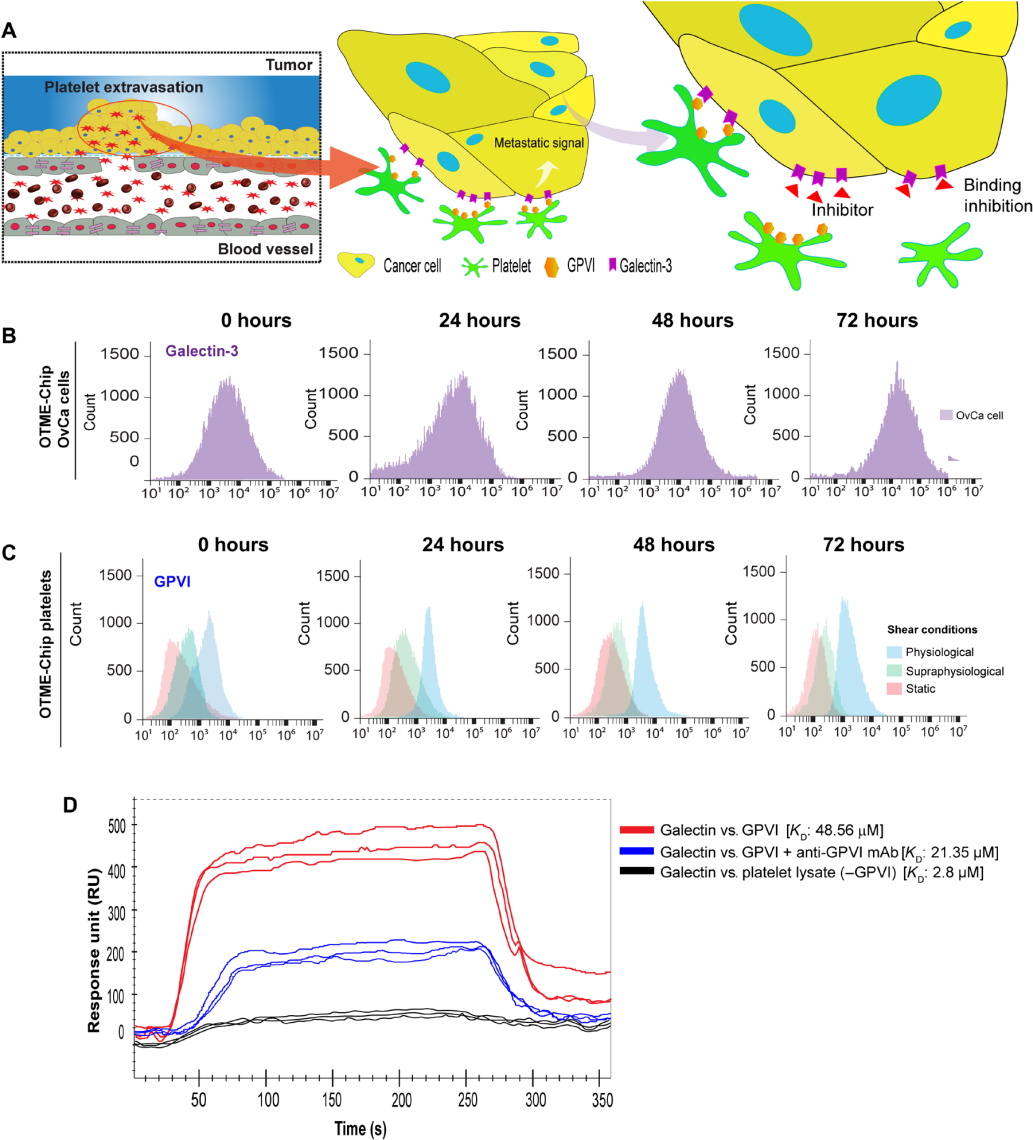
Platelet GPVI expression is shear dependent and it binds to tumor galectin-3 in OTME-Chip. (**A**) Infographic describing platelet extravasation into the tumors inside the chip and platelet GPVI interaction with ovarian tumors through binding to galectin-3, which results in a prometastatic and chemoresistive tumor microenvironment. Therefore, GPVI inhibitors may be a therapeutic target to arrest ovarian cancer metastasis. Representative flow cytometry tracings at variable time points from cells recovered from the OTME-Chip, corresponding to (**B**) galectin-3 expression on the surface of ovarian cancer cells and (**C**) GPVI expression on the surface of extravasated platelets. (**D**) Surface plasmon resonance tracings (*n* = 3 independent experiments) show strong binding affinity between GPVI and galectin-3 proteins isolated from the platelets and cancer cells (red) against platelets exposed to anti-GPVI monoclonal antibody (mAb) (blue). GPVI-free platelet lysate shows no binding with galectin-3 (black). Binding affinity (*K*_D_) values are indicated in brackets.

In several cancers, including ovarian cancer, the expression of galectins is up-regulated and induces platelet adhesion and hyperactivity (*12*, *24*, *25*). Adhered and activated platelets, in turn, prime cancer cells for metastasis. Tumor galectin-3 specifically contains a collagen-like domain. On the other hand, the collagen receptor on platelets, GPVI, regulates platelet activation under shear (*26*). Therefore, we examined whether OTME-Chip may reveal the contribution of GPVI–galectin-3 binding to the platelet–cancer cell interaction and its metastatic consequences under shear (Fig. 2A). We first examined the expression and activity of galectin-3 over the course of our 72-hour timeline of exposure of cancer cells to extravasated platelets and found that galectin-3 expression was conserved and did not change over time (Fig. 2B).

Next, we analyzed the expression of platelet GPVI within OTME-Chip. Since platelet GPVI is known to be shear sensitive (*27*), we decided to compare platelet GPVI expression under variable shear. We found that when we perfused the platelets at a typical physiological shear (~1 dyne cm^−2^), GPVI expression was high, but, in contrast, both static condition and perfusion at a supraphysiological shear (~5 dyne cm^−2^) resulted in a diminished GPVI expression (Fig. 2C). Our data suggest that at low physiological shear, GPVI–galectin-3 interactions may be potent because of the high availability of GPVI.

Thereafter, to evaluate platelet GPVI binding with tumor galectin-3, immediately after exposing the platelets to shear within the OTME-Chip and extravasation, we isolated and purified the GPVI protein from the platelets and galectin-3 from the ovarian cancer cells. A rapid increase in the binding signal was detected when platelet-derived GPVI protein was perfused on a surface plasmon resonance chip coated with galectin-3 protein isolated from the tumors (Fig. 2D). In contrast, the binding was absent when a GPVI-free platelet lysate (immune pulled down) was used and substantially diminished when an inhibitory anti-GPVI monoclonal antibody was perfused along with GPVI. In summary, our analyses suggest that platelet GPVI binds to tumor galectin-3.

### Consequences of GPVI and galectin-3 interaction in ovarian cancer assessed with OTME-Chip

**Fig. 3:**
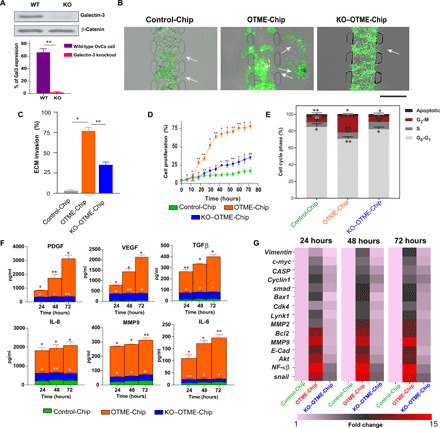
Platelet GPVI promotes ovarian metastasis through galectin-3 binding to cancer cells in OTME-Chip. (**A**) Western blot and corresponding densitometry analyses shows highly reduced galectin-3 (Gal3) protein in the KO cancer cells against wild-type (WT) controls. (**B**) Representative fluorescence microscopy images showing cancer cell (green) invasion (marked by arrows) into hydrogel ECM due to extravasated platelets (yellow). Scale bar, 200 μm. (**C**) Bar graph showing the quantification of ECM invasion in Control-Chip, OTME-Chip, and KO–OTME-Chip at 48 hours. Analysis of (**D**) cancer cell proliferation, (**E**) flow cytometry–based cell cycle phases, (**F**) excreted growth factors (top) and cytokines (bottom) over time, and (**G**) real-time polymerase chain reaction (RT-PCR) heatmaps showing expressional alteration of cell proliferation and metastasis regulatory genes in Control-Chip, OTME-Chip, and KO–OTME-Chip. *P < 0.05 and **P < 0.01; *n* = 3 individual experiments; error bars are means ± SEM. One-way analysis of variance (ANOVA) is done followed by Dunnett’s multiple comparisons test.

We then investigated the possibility that the GPVI–galectin-3 interaction may serve as a mediator for platelet-promoted tumor metastasis in ovarian cancer. To test this hypothesis, we established an ovarian cancer cell line with galectin-3 knocked out (KO-g3) using the CRISPR-Cas9 editing method (Fig. 3A). We compared the parameters that govern cancer cell metastasis (ECM invasion, proliferation, cytokine profile, and transcriptional readouts) in OTME-Chips that were either composed of original ovarian cancer cells (OTME-Chip) or galectin-3 knocked down cancer cells (KO–OTME-Chip). We found rapid hydrogel ECM invasion by the wild-type ovarian cancer cells when cocultured with extravasated platelets (OTME-Chip) relative to controls devoid of platelets (Control-Chip). However, we observed diminished ECM invasion in KO–OTME-Chips in the presence of extravasated platelets (Fig. 3, B and C). Cancer cell proliferation increased in OTME-Chips relative to controls during 40 to 72 hours of extravasated platelets and tumor interaction, and proliferation was found diminished in galectin-3 KO–OTME-Chips (Fig. 3D). We observed this OvCa cell proliferation to be proportional to their ECM hydrogel invasion within our observation timeline.

G_2_-M extension and reduced apoptosis are a hallmark of metastasis of several carcinomas (*28*, *29*). Cell cycle analysis of these OTME-Chips showed rapid alterations in cell cycle phases. A prominent G_2_-M phase extension and reduced apoptosis were observed in wild-type ovarian cancer cells due to platelets (OTME-Chip) as compared to their platelet-less counterpart (Control-Chip), and this extended G_2_-M was found to be reduced in KO-g3 cancer cells even in the presence of platelets (KO–OTME-Chip; Fig. 3E). We examined the effluents obtained from the tumor chambers of OTME-Chips and found a time-dependent increase in the concentration of growth factors platelet-derived growth factor (PDGF), vascular endothelial growth factor (VEGF), and transforming growth factor–β (TGFβ) and cytokines IL-8, matrix metalloproteinase 9 (MMP9), and IL-6 in effluents due to extravasated platelets that were significantly reduced in KO–TME-Chips (Fig. 3F).

We also hypothesized that GPVI–galectin-3 interaction eventually leads to the activation of the downstream nuclear factor κβ (NF-κβ) pathway in cancer cells. This NF-κβ signaling acts in combination with TGFβ/SMAD pathway to trigger tumor metastasis and rapid proliferation. Gene expression analysis of TME-Chips revealed a rapid up-regulation in the expression of proliferation and metastasis genes in the wild-type ovarian cancer cells due to extravasated platelets compared to KO-g3 cancer cells and platelet-less controls (Fig. 3G). A time-dependent rapid increase in the expression of *BCL2*, *MMP9*, *E-CADHERIN*, *AKT*, *NF-k*β, and *SNAIL* and a moderate increase in *VIMENTIN*, *C-MYC*, *CASP*, *CYCLIN1*, *SMAD*, *BAX*, *CDK4*, *LYNK1*, and *MMP2* similar to hallmark cell proliferation and metastasis regulator genes are observed in cancer cells over the coculture time due to platelets. These data strengthen the characterization of GPVI–galectin-3 interaction as an initiating factor behind the transcriptional signaling that is necessary for the proliferation and metastasis of ovarian cancer.

### OTME-Chip predicts that pharmaceutical inhibition of GPVI arrests metastasis and supports chemotherapy

**Fig. 4:**
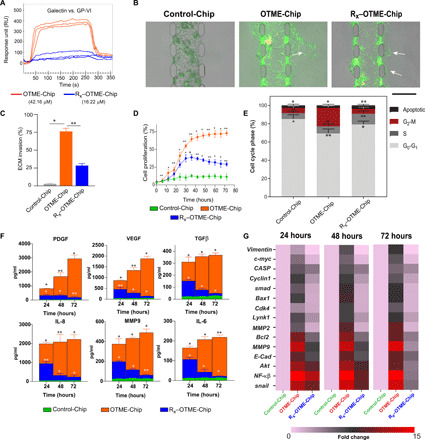
Revacept (GPVI inhibitor) arrests ovarian metastasis in OTME-Chip. (**A**) Surface plasmon resonance tracings (*n* = 3 independent experiments) show strong binding affinity between GPVI and galectin-3 proteins isolated from the platelets and cancer cells (red) against platelets exposed to Revacept (blue). Binding affinity (*K*_D_) values indicated in brackets. (**B**) Representative fluorescence microscopy images showing cancer cell (green) invasion into hydrogel ECM due to extravasated platelets (yellow). Scale bar, 200 μm. (**C**) Bar graph showing the quantification of ECM invasion in Control-Chip, OTME-Chip, and R_x_–OTME-Chip. Analysis of (**D**) cancer cell proliferation, (**E**) flow cytometry–based cell cycle phases, (**F**) excreted growth factors (top) and cytokines (bottom) over time, and (**G**) RT-PCR heatmaps showing expressional alteration of cell proliferation and metastasis regulatory genes in Control-Chip, OTME-Chip, and R_x_–OTME-Chip. *P < 0.05 and **P < 0.01; *n* = 3 individual experiments; error bars are means ± SEM. One-way ANOVA is done followed by Dunnett’s multiple comparisons test.

We followed up with investigating the potential consequences of pharmaceutical targeting of GPVI on platelets in cancer. There are currently no US Food and Drug Administration (FDA)–approved anti-GPVI drugs in the market. However, Revacept is an inhibitor that has been successfully evaluated against collagen-mediated platelet adhesion in phase 1 clinical trials of atherosclerosis and stroke (*30*). Revacept inhibited the platelet GPVI and tumor galectin-3 interaction in a colon carcinoma cell line (*12*). This drug is a fusion protein consisting of the Fc part of human immunoglobulin G (IgG), a short hinge region, a specific linker sequence, and the extracellular part of human platelet GPVI with a molecular mass of 150 kDa. We hypothesized that the specific inhibition of platelet GPVI with antiplatelet drug Revacept would arrest the tumor’s metastatic potential within our in vitro OTME-Chip. Subsequent to platelet perfusion in OTME-Chip and extravasation of platelets into the ovarian cancer cells, we exposed the tumors to Revacept at its clinically relevant concentration (40 μg/ml) (*12*). Thereafter, these chips were analyzed for 3 days, as described in our Materials and Methods (fig. S1). We found that Revacept-treated OTME-Chips (R_x_–OTME-Chips) exhibited significantly reduced GPVI–galectin-3 binding kinetics (Fig. 4A). Revacept significantly reduced ECM invasion and proliferation rate of the cancer cells, suggesting that GPVI inhibition was also preventing the consequence of GPVI–galectin-3 interaction (Fig. 4, B to D). To confirm that our results are not dependent on A2780 cancer cells alone, we also tested a moderately invasive ovarian cancer cell line OVCAR3 (*31*, *32*) and again observed an increased cancer cell invasion due to platelets but inhibited by Revacept (fig. S2).

Cell cycle analysis revealed a reduced G_2_-M phase in cancer cells from R_x_–OTME-Chips compared to untreated controls (Fig. 4E). Further, Revacept reduced the growth factors (PDGF, VEGF, and TGFβ) and cytokines (IL-8, MMP9, and IL-6) (Fig. 4F). The transcriptional analysis of cancer cells obtained from the chips showed that the platelet-induced up-regulation of metastasis and cell proliferation genes declined rapidly over time in R_x_–OTME-Chips (Fig. 4G).

**Fig. 5:**
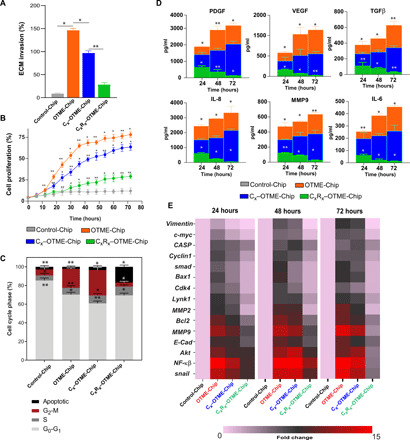
Revacept (GPVI inhibitor) decreases chemoresistance in OTME-Chip. Analysis of (**A**) ECM invasion, (**B**) cancer cell proliferation, (**C**) flow cytometry–based cell cycle phases, (**D**) excreted growth factors (top) and cytokines (bottom) over time, and (**E**) RT-PCR heatmaps showing expressional alteration of cell proliferation and metastasis regulatory genes in Control-Chip, OTME-Chip, C_x_–OTME-Chip, and C_x_R_x_–OTME-Chip. *P < 0.05 and **P < 0.01; *n* = 3 individual experiments; error bars are means ± SEM. One-way ANOVA is done followed by Dunnett’s multiple comparisons test.

Furthermore, since platelets have been shown to promote tumor chemoresistance (*33*, *34*), we examined the effect of GPVI–galectin-3 interaction and blocking this interaction by Revacept on chemoresistance in ovarian cancer cells. We treated our OTME-Chips with either no drug (OTME-Chip) or anticancer drug cisplatin alone near its clinically relevant concentration (6 μg/ml) (C_x_–OTME-Chip) (*35*) or cisplatin compounded with Revacept (C_x_R_x_–OTME-Chip). We found that cisplatin alone had a modest effect, whereas the cisplatin-Revacept combination had a significant effect in reducing tumor invasiveness (Fig. 5A), cell proliferation (Fig. 5B), G_2_-M phase (Fig. 5C), and concentration of growth factors and cytokines (Fig. 5D). Last, transcriptional analysis of cancer cells obtained from the chips showed that the platelet triggered up-regulation of metastasis and cell proliferation genes altered overtime modestly when cisplatin alone is included within OTME-Chips (C_x_–OTME-Chip); however, Revacept significantly attenuated this overexpression **(**C_x_R_x_–OTME-Chip; Fig. 5E). Together, our OTME-Chip’s capability to assess anticancer drugs systematically provides us these preclinical datasets, which suggest that GPVI inhibition could be a potential strategy against ovarian cancer metastasis and chemoresistance.

### Validation of cancer cell–platelet-drug cross-talk within OTME-Chip with next-generation RNA-seq

**Fig. 6:**
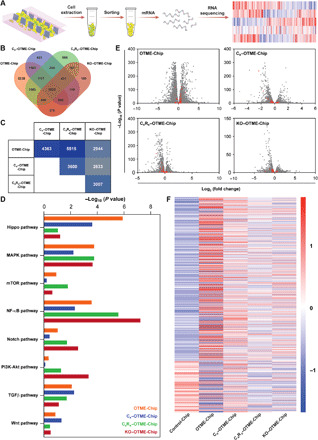
RNA-seq and differential gene expression analysis reveals the efficacy of antiplatelet therapy to attenuate tumor-promoting pathways. (**A)** Schematic of cancer cell isolation, cell sorting, and mRNA isolation process used in the study. Samples were then assessed for quality and processed for RNA sequencing. (**B**) Figure shows Venn diagram of differentially expressed genes among all groups. RNA sequencing and differential gene expression analysis revealed enrichment of 12,452, 5566, 6909, and 3821 genes in the OTME-Chip, C_x_–OTME-Chip, C_x_R_x_–OTME-Chip, and KO–OTME-Chip, respectively, with respect to Control-Chip. (**C**) Group-wise comparison of common genes for all combinations. OTME-Chip and C_x_R_x_–OTME-Chip had the highest number common genes (~5500 genes). (**D**) KEGG pathway clustering revealed differential presence of EMT regulatory pathways among groups relative to Control-Chip (*P* < 0.05). (**E**) Volcano plots showing differentially expressed genes for all groups relative to Control-Chip (black dots). Red dots signify genes regulating EMT and metastasis. (**F**) Heatmap showing row-scaled *z* scores of ~900 genes belonging to the pathways as depicted in (E). For each group, *n* = 3 number of samples were sequenced.

We have recently shown that a collaboration of organ-chip technology with RNA-seq analysis is a powerful tool to validate preclinical studies and derive unbiased clinical predictions (*36*). Therefore, to solidify our insights into the platelet-tumor signaling nexus and the therapeutic effect of Revacept, we performed transcriptomic profiling of different chip-derived tumors through RNA-seq (Fig. 6A). Through RNA-seq and differential gene analysis, we detected expression of 12,452 genes in the OTME-Chip relative to Control-Chip. Similarly, we detected expression of 5566, 6909, and 3821 genes in the C_x_–OTME-Chip, C_x_R_x_–OTME-Chip, and KO–OTME-Chip, respectively, relative to Control-Chip (Fig. 6B). The reduction in differentially expressed genes under the different drug-treated and KO conditions suggests that the drug treatments were attenuating the transcriptomic expression and transformed the physiological behavior of the chips close to untreated controls. Among the different chip conditions, there were 15 groups of genes that were either unique to each condition or were common between chip conditions for the different permutations (Fig. 6B). Comparing genes common to two chip conditions at a time, we observed that the OTME-Chip and C_x_R_x_–OTME-Chip conditions had the highest number of common genes (~5500; Fig. 6C). In addition to the NF-κB and TGFβ/SMAD pathways that have been elucidated before (*5*, *7*, *8*) and in this work (Fig. 3), the RNA-seq analysis allowed us to investigate other signaling pathways that were up-regulated under the different chip conditions via Kyoto Encyclopedia of Genes and Genomes (KEGG) pathway clustering. When compared to the platelet-free tumors (Control-Chip), we observed that in addition to the already known NF-κB and TGFβ/SMAD pathways, epithelial-mesenchymal transition (EMT) and metastasis regulatory signaling pathways such as Hippo (KEGG:04390), MAPK (mitogen-activated protein kinase) (KEGG:04010), mTOR (mechanistic target of rapamycin) (KEGG:04150), Notch (KEGG:04330), phosphatidylinositol 3 kinase-protein kinase B (PI3-Akt) (KEGG:04151), and wingless-related integrated site (Wnt) (KEGG:04310) pathways were up-regulated (Fig. 6D). This result suggests that targeting these other pathways could further open new avenues toward the development of anticancer therapeutics. To further visualize the efficacy of different treatments, we generated volcano plots and highlighted genes belonging to the aforementioned signaling pathways (Fig. 6E). As expected, both the chemotherapy drug cisplatin and the antiplatelet Revacept reduced the expression of these genes. This down-regulation in expression was also confirmed by generating heatmaps, showing holistic differences in expression profiles of the different chip conditions (Fig. 6F). OTME-Chip had a complementary expression profile compared to the Control-Chip (Fig. 6F). Consequently, we observed that cisplatin therapy down-regulated the expression of these pathways and that a combination therapy of cisplatin and Revacept further reduced their expression. This down-regulation in the C_x_R_x_–OTME-Chip was close to the KO-g3 cancer cells available from KO–OTME-Chip, where the GPVI–galectin-3 interaction was disturbed via KO. This result unbiasedly validates that GPVI–galectin-3 interaction is pivotal in platelet-mediated tumor promotion and establishes the therapeutic efficacy of antiplatelet drug Revacept against the progression of cancer with this preclinical platform.

## DISCUSSION

Although several other groups have pioneered the creation of bioengineered models of tumors alone, tumor-vessel constructs, and tumor-ECM constructs, the primary focus of these studies did not include examining platelet-tumor interactions and consequences (*22*, *37*–*41*). Notably, platelets are one of the first blood cells to interact with cancer cells, and it is increasingly getting appreciated clinically that thrombocytosis is associated with a poor prognosis in several cancers (*42*, *43*). Specifically, in ovarian cancer, animal models convincingly support that platelets extravasate into the OTME and increase proliferation and EMT in ovarian cancer cells (*4*, *5*, *33*). In the current study, we built upon our prior expertise in leveraging organ-chip technology to integrate blood cell function into modeling processes in cancer. Here, we created a novel OTME-Chip that demonstrates a biomimicry of the consequences of the platelet–cancer cell interaction in ovarian cancer that is hard to achieve via conventional in vivo or in vitro models. The addition of hydrogel compartments adjacent to the cancer cell–vessel interface within the chip permitted time-lapse visualization of perfused platelets within the vessel, their extravasation through the endothelium, and consequential cancer cell invasiveness that was not previously obtained through ectopic in vivo experiments. Furthermore, isolating cancer cells from the chip during different time points of platelet perfusion have allowed us to identify the platelet-mediated increase in tumor proliferation and specific alterations in cancer cell cycle phases. Although this study is limited to ovarian cancer, this platform can be expanded to other cancer models and include more complex formulations of matrices and interstitial microenvironment.

We demonstrated that GPVI is a critical mediator in the adhesion of extravasated platelets to cancer cells. The counter receptor for GPVI is galectin-3, which is expressed on cancer cells. We showed that platelet GPVI expression is shear dependent, and, therefore, its interaction with the tumors could be dependent on the hemodynamic state. Replacing wild-type cancer cells with KO-g3 cells in OTME-Chip identified the key regulatory role of GPVI–galectin-3 interaction in metastasis. Our in vitro results are supported by several clinical observations and animal studies. For example, overexpression of galectin-3 increased tumor burden in A2780 ovarian cancer xenografted mice. Increased expression of galectin-3 has also been detected in advanced stages in patients with ovarian cancer (*44*). Increased levels of soluble GPVI have also been reported in plasma samples (*11*). GPVI blockade inhibited lung, colon, and breast cancer metastasis in mouse models (*11*, *12*, *45*). Studies in colorectal and breast cancer have described an increase in growth factors and cytokines in the TME after platelet extravasation into the tumors, and this has been predicted due to GPVI–galectin-3 interaction (*7*, *10*, *12*). In ovarian cancer, a similar elevation of growth factors and cytokines in TME is observed due to extravasated platelets (*5*). In addition, the extravasated platelets have been identified to cause proliferation of tumors via TGFβ/SMAD pathway, but the regulation of GPVI–galectin-3 interaction on activation of NF-κβ signaling has not been explored. Analysis of effluents from tumor microenvironment collected from the chips during the hours of platelet-tumor interaction by transcriptomics and multiplexing methods detected alterations in gene expression and protein profile. This identified the up-regulation of a series of metastasis triggering growth factors and cytokines due to GPVI–galectin-3 interaction.

Antiplatelet therapy has been tested in several clinical trials of cancer in the past (*46*). For example, acetylsalicylic acid or aspirin has been suggested to reduce platelet aggregation on tumors and inhibit metastasis. In an in vitro study, aspirin and P2Y_12_ inhibition attenuated platelet-induced ovarian cancer cell invasion (*47*). However, these drugs have a major drawback that they can increase bleeding risk in patients. Therefore, antiplatelet therapeutics that may be antimetastatic but do not alter hemostasis is an important unmet need. Platelet GPVI is an excellent target since it is known to be antithrombotic but does not cause bleeding (*48*). With our OTME-Chip, we have provided preclinical evidence that Revacept inhibited platelet-cancer cell interactions and its consequences and, therefore, is a potential drug to be advanced into clinical trials of ovarian cancer. Last, we showed that the GPVI–galectin-3 interaction induces chemoresistance in cancer cells, and Revacept can inhibit this effect. The inhibitory effect of Revacept treatment on cancer cell invasion independently and in combination with cisplatin was also verified in detail through the RNA-seq analysis. Therefore, our study has a translational value in the context that it motivates Revacept to be tested in a clinical trial against ovarian and other cancers.

Although, in this study, we focused on cancer metastasis triggered by the extravasated platelets, it is possible that blood immune cells such as tumor-associated macrophages, myeloid-derived suppressor cells, and regulatory T cells can also support tumor metastasis by forming a cumulative signaling along with platelets that remodel the tumor microenvironment (*49*–*51*). These immune cells could be included in this OTME-Chip study to identify their influence on tumors and metastasis. We did not include pericytes while forming the vascular tissue inside the chip, and pericytes can potentially exert influence on platelet extravasation (*52*). We also did not perfuse whole blood in the vascular chamber because it is very difficult to maintain blood fluidity in vitro for a prolonged period. Last, unlike some other tumor models where cancer cells are fully embedded within the matrix, cancer cells in our model were seeded over the matrix to prevent nonspecific interactions between platelets and cancer cells and to enable robust visual and molecular analytics. This relative simplicity and a provision to do analytics is a strength of our OTME-Chip, which is also not radically different from its prior versions (*20*) and consistent with prior models (*39*). Overall, this preliminary device design is a suitable proof of concept that highlights out OTME-Chip as a powerful tool in the investigation of blood cell influence on cancer and metastasis. An interesting future opportunity this platform offers is to also include the interaction of platelets with circulating tumor cells that may possibly disseminate from the solid tumor into the vascular chamber of this chip.

In conclusion, we demonstrated that complex interaction between the tumors and blood platelets and the resulting metastatic process could be modeled in a bioengineered tumor microenvironment with vascular components on a chip. We uncovered that this platform could be used in the identification of complex tumor–blood cell interactions, metastatic signaling, and the evaluation of drugs with an integrated genomic and proteomic approach. In addition, the scope of application of desired anticancer or antiplatelet drugs individually or in combination also makes our device a potential platform to perform drug evaluation studies against platelet-triggered metastasis and chemoresistance of cancer. Opportunity to include ECM in the chip also added additional capability to investigate the tissue invasiveness and the invasion behavior of tumors due to platelets under different conditions. We expect that this OTME-Chip technology could further be advanced toward personalized cancer medicine by integrating induced pluripotent stem cells derived from malignant tumors and other patient-derived blood or immune cells, where we can dissect the interaction signaling underlying metastasis. In summary, our human OTME-Chip model may offer a potential platform for studying the interaction between blood cells and cancer cells, acquiring functional readouts, and preclinical data of different antimetastasis drugs before initiating large animal studies and human clinical trials.

## MATERIALS AND METHODS

### Cell lines

HOMECs (ScienCell Research Laboratories) were cultured in endothelial cell medium (ECM; catalog no. 1001), and ovarian carcinoma A2780 cells (Sigma-Aldrich, catalog no. 93112519) were cultured in RPMI 1640 medium. Each was supplemented with penicillin (100 U ml^−1^) and streptomycin (100 μg ml^−1^). Tumor KO-g3 A2780 cells were developed using CRISPR-Cas9 methods and selected on puromycin (1 mM). Single guide RNAs (gRNAs) targeting the exon 3 of galectin-3 were designed for the KO study. The designed guides were screened in silico for off-target activity using the CRISPR search with mismatches, insetions and/or deletions (COSMID) webtool (*53*), and the gRNA (CATGATGCGTTATCTGGGTC) with the least number of off targets was selected for the KO experiments. The guide was delivered as a ribonucleoprotein complex with Cas9 to the A2780 cell line using an optimized nucleofection protocol.

### Human platelets

Platelets were isolated from fresh human donor blood samples following previously mentioned protocol (*20*) using acid citrate dextrose buffer and Hepes-Tyrode’s buffer to preserve their cytoskeletal activity. Platelets were tagged using phycoerythrin-conjugated CD41 monoclonal antibody (1:200 dilution; Thermo Fisher Scientific, CAS-VIPL3) before perfusion into the device (~200 × 10^3^ platelets μl^−1^). Blood samples were obtained via phlebotomy according to the policies of the U.S. Office of Human Research Protections and approved by the Texas A&M University Institutional Review Board (ID: IRB2016-0762D).

### Drugs

Cisplatin (Thermo Fisher Scientific, USA) and Revacept (advanceCOR GmbH, Germany) were dissolved in dimethyl sulfoxide and phosphate-buffered saline (PBS)/4% mannitol/1% sucrose, respectively, to prepare master stocks (1 mg/ml). These were further diluted in RPMI 1640 medium to obtain respective working concentrations of cisplatin (6 μg ml^−1^) and Revacept (40 μg ml^−1^). They were perfused into the chip tumor chamber individually or in combination in the presence or absence of extravasated platelets.

### Design and fabrication of the microfluidic OTME-Chip

OTME-Chips were adapted from previously reported OvCa-Chips (*20*), where the top portion of the device was changed from a single microchannel (1 mm in length by 1 mm in wide by 100 μm in height) to three parallel microchannels. The three microchannels were separated by hexagonal micropillars instead of a continuous PDMS wall, allowing for the formation of hydrogel fluid barriers that supported cell invasion. Each micropillar had dimensions of 250 μm by 100 μm by 100 μm and was equally spaced, leaving 100 μm between each micropillar. Drawings of the device were made using SOLIDWORKS 2019, from which photomasks were printed. Master molds were made using traditional soft lithography procedures (*54*). PDMS slabs and porous membrane were casted and cured, assembled, and processed according to previously developed protocols (*20*).

Pregelled hydrogel solutions were prepared on ice by neutralizing rat tail collagen I (3.57 mg ml^−1^) (Corning) with 1 N of NaOH before diluting to 2.5 mg ml^−1^ with cell culture medium. Pregelled (5 μl) hydrogel solution were injected into each ECM channel, and the device was stored in an incubator at 37°C for 1 hour for gel solidification. After gelation, hydrogel channels were hydrated by injecting a mixture of rat tail collagen type I (100 μg ml^−1^; BD Biosciences) and fibronectin (30 μg ml^−1^; BD Biosciences) inside the tumor and vessel chambers of chip and incubated at 37°C for at least 2 hours. This step also prepared cellular compartments for cell seeding. Before loading cells into their respective chambers, a wash with one-time PBS was performed to remove unbound residual collagen. HOMECs and A2780 OvCa cells were harvested and centrifuged, their supernatant removed, and pellets resuspended in respective fresh culture medium before loading into devices. HOMEC cell suspension (5 × 10^5^ cells ml^−1^) was injected inside the vessel chamber and incubated at 37°C by following previous protocols (*20*) to obtain a confluent microvessel of HOMECs. After the microvessel was formed, the collagen-coated upper tumor chamber of the chip was seeded with cancer cell suspension (5 × 10^5^ cells ml^−1^) and cultured for 24 hours to obtain a confluent monolayer. Platelets were perfused inside the vascular chamber using a syringe pump. The shear stress reported is at the wall that was calculated from the Navier-Stokes’ equation applicable for simple rectangular channels

$$\tau_{w}=\frac{6\eta Q}{h^{2}w}$$

where τ_w_ is wall shear stress, η is dynamic viscosity of fluid, *Q* is the flow rate, and *h* and *w* are the microfluidic channel’s height and width, respectively. After platelet extravasation, the cancer cells inside the tumor chamber were cultured under a continuous flow of RPMI 1640 medium for another 3 days (~1 dyne cm^−2^). Tumor chamber medium effluent was collected from the devices at 24, 48, and 72 hours. Cancer cells and platelets were isolated from the tumor chamber using Accutase treatment, and the CD41-tagged platelets were subsequently pulled down of from the cancer cell–platelet mixture using MojoSort human anti-CD41 magnetic nanobeads (BioLegend, USA) on a magnetic separator provided by the manufacturer. The magnetically separated platelet and tumor fractions were washed and kept for further downstream assays.

### Immunostaining and fluorescence-activated cell sorting

Platelets and cancer cells isolated from the device were washed by centrifugation [300 relative centrifugal force (RCF) for 5 min] and resuspended in one-time PBS, followed by blocking with 2% bovine serum albumin (BSA) for 10 min and fixing with 2% paraformaldehyde for 15 min at room temperature. Fixed cells were permeabilized using 0.1% Triton X-100 for 5 min before immunostaining. Platelets were immunostained for GPVI surface marker using antihuman GPVI (Sigma-Aldrich, catalog no. ABS446) primary antibody, followed by incubation with appropriate fluorescent secondary antibody as per experimental need. Fixed and permeabilized cancer cells were stained for galectin-3 protein using antihuman galectin-3 primary antibody (Thermo Fisher Scientific, catalog no. A3A12) and compatible fluorescent-tagged secondary antibody before flow cytometry experiments. All flow cytometric analyses were performed using a BD Accuri C6 flow cytometer (BD Biosciences, USA), and data were processed using CellQuest Pro software.

### Cell invasion

Cancer cells were live stained with PKH67 cytotracker-green (Sigma-Aldrich, USA) before seeding into the device. After extravasation, the ovarian cancer cells were incubated at 37°C with continuous perfusion of medium in tumor chamber for 3 days. Invasion dynamics of live-stained cancer cells (green) into the chip ECM chamber hydrogel was visualized using fluorescence microscopy (ZEISS Axio Observer; LD Plan Neofluar 10×, numerical aperture 0.4). Snapshots were taken at every 12 hours of interval with an exposure time of 200 ms. Images were analyzed and processed using software ZEN 2.3 lite (ZEISS). Measurement of cancer cell invasion through the hydrogel was performed using cell counter plugins in ImageJ. The ECM invasion of cancer cells was calculated as the ratio of area occupied by invaded cancer cells in ECM chamber versus the total area of ECM chamber (*55*).

### Cancer cell proliferation assay

Isolated cancer cells were immunostained with monoclonal anti-Ki67 antibody Alexa Fluor 488 conjugate (Abcam). The fluorescence intensity was then measured with a plate reader using excitation/emission wavelengths of 488/519 nm. Platelet-free cancer cells obtained from other chips during similar time points were kept as control. Proliferation rate was measured as a percentage ratio of fluorescent intensities of experimental and control cells.

### Cell cycle analysis

Cancer cells isolated via Accutase treatment from the chip were fixed in 70% ethanol at 20°C for 6 hours and permeabilized. Cells were then incubated with propidium iodide (100 μg ml^−1^) (Sigma-Aldrich) and ribonuclease A (50 μg ml^−1^) in the dark for 30 min at 37°C. Flow cytometry was performed on a BD Accuri C6 flow cytometer (BD Biosciences, USA), and cell cycle analysis was performed using CellQuest Pro software.

### Growth factors and cytokines

Growth factors and cytokines present in OTME-Chip tumor chamber medium effluents were measured using multiplex (xMAP) magnetic bead–based technology provided by Millipore. Medium effluents collected from tumor chamber during different time points were analyzed for cytokines and growth factors using a MILLIPLEX MAP human cytokine/chemokine magnetic bead panel kit (Millipore, CAS-HCYTOMAG-60K) by following previously established protocols. Assays were run in a precalibrated Luminex MAGPIX reader. The mean fluorescent intensity of the magnetic beads correlating with the concentration (picograms per milliliter) of respective cytokines and growth factors was calculated using xPONENT 4.2 software, and data analysis was done with MILLIPLEX Analyst 5.1 software (Millipore). The concentration of cytokines obtained in each sample was normalized with respect to controls, and the assays were run in triplicate. Cytokine data for each group were normalized to corresponding cell number, respectively.

### Quantitative real-time polymerase chain reaction

Cancer cell total RNA was isolated using the Arcturus PicoPure RNA Isolation Kit (Thermo Fisher Scientific, CAS-KIT0214) specifically designed to recover high-quality RNA consistently from fewer cells. Cells isolated from the chip were washed in prescribed buffer (0.9 ml of one-time PBS/10% BSA and 0.1 ml of 0.5 M EDTA), and total cellular RNA was isolated using the manufacturer’s protocol. Isolated RNA was treated with deoxyribonuclease, and its quality was checked to be standard 260/280 of >1.5 with spectrophotometry before 0.5 μg of RNA was processed further for the preparation of cDNA using the ProtoScript First Strand cDNA Synthesis kit (New England Biolabs). The cDNA prepared was further used for the subsequent quantitative real-time polymerase chain reactions (qRT-PCRs) using SYBR Green master mix (Applied Biosystems SYBR Green Select PCR Master Mix) and gene-specific primers. PCRs for respective genes were performed in qRT-PCR system (Thermo Fisher Applied BioSystems Quantstudio 3) keeping glyceraldehyde-3-phosphate dehydrogenase as an internal control. Gene expression was analyzed by quantifying the relative fold changes in mRNA levels of individual genes using comparative cycle threshold (CT)/ΔCT method.

### Western blot

Galectin-3 knockdown in cancer cells was verified by Western blot assay. Briefly, cell lysates were prepared from equal amount of wild-type and KO A2780 cells, and protein extraction was performed using spin columns. Equal amount of protein (20 μg) from harvested cells was loaded onto 10 to 15% (w/v) polyacrylamide gels and separated by SDS–polyacrylamide gel electrophoresis, followed by transfer to polyvinylidene difluoride membrane. After transfer, the membrane was incubated with anti–galectin-3 antibody (1:10,000; Thermo Fisher Scientific, CAS-A3A12) and anti–β-actin antibody (1:10,000 dilution; Thermo Fisher Scientific, CAS-PA1-183), followed by incubation with horseradish peroxidase–conjugated anti-rabbit IgG (1:5000 dilution; Abcam, CAS-ab6721) secondary antibody. The protein bands were then visualized with chemiluminescence (Bio-Rad, USA), and densitometry quantification was performed using ImageJ analysis software.

### Surface plasmon resonance

Physical interaction between platelet surface GPVI and tumor galectin-3 was determined via level-free analysis using surface plasmon resonance method (Nicoya Life, USA). Galectin-3 protein was pulled down from cancer cell and platelet lysates using biotinylated anti–galectin-3 monoclonal antibody and anti-GPVI monoclonal antibody, respectively. The proteins were diluted in HBS-EP-EDTA running buffer [0.01 M Hepes, 0.15 M NaCl, and 3 mM EDTA (pH 7.4)]. Streptavidin-coated chips purchased from Nicoya Life were used to immobilize biotinylated galectin-3 by injecting the protein into the device for 5 min using a flow rate of 20 μl min^−1^, thus immobilizing biotinylated galectin-3 on the streptavidin-coated chip. Successful immobilization was confirmed by observation of an increase of ~50 response units (RU) in the sensorgram signal. Purified GPVI (100 μl) was injected at a flow rate of 20 μl min^−1^ over the galectin-3–immobilized chip, and the resonance unit change in the sensorgram signal was recorded. For inhibition studies, Revacept (40 μg ml^−1^) was added with purified GPVI and injected into the device. The data obtained were analyzed by curve fitting of the Langmuir binding isotherm with the software.

### RNA-seq and analysis

RNA from the chips were isolated according to the method described earlier. Isolated mRNA samples were then analyzed using the NextSeq 500 platform (Illumina), and sample preparation was performed using TruSeq RNA sample preparation with a paired-end read length of 2 × 75 bases (Molecular Genomics Workspace, Texas A&M University, College Station, TX). Then, we used hierarchical indexing for spliced alignment of transcripts 2 (HISAT2) to splice align the reads to latest Ensembl release 102 human genome/transcriptome (GRCh38.p13). To generate raw counts from alignment files (SAM), we used the Bioconductor package Subread. Differentially expressed genes were then evaluated for all groups using DESeq2 package in R. The cutoff to determine significant genes in all groups were false discovery rate–adjusted *P* value (*q* value) of <0.05. Last, KEGG pathway analysis was performed using the online database Database for Annotation Visualization and Integrated Discovery (v6.8) and visualized using Cytoscape and ClueGO (*56*).

### Statistical analysis

All results are presented as means ± SEM, unless otherwise noted. Data were analyzed using one-way analysis of variance (ANOVA), followed by Dunnett’s multiple comparisons test using GraphPad Prism (GraphPad Software, San Diego, CA, USA). *P* < 0.05 was considered as statistically significant.

## SUPPLEMENTARY MATERIALS

Supplementary material for this article is available at http://advances.sciencemag.org/cgi/content/full/7/30/eabg5283/DC1

## Appendix

View/request a protocol for this paper from *Bio-protocol*.
